# Relationship of Nutritional Status and Spirometric Parameters in Children with Bronchial Asthma

**DOI:** 10.17691/stm2020.12.3.02

**Published:** 2020-06-28

**Authors:** R.N. Khramova, E.V. Tush, A.A. Khramov, D.Yu. Ovsyannikov, K.S. Popov, I.V. Dolbin, O.V. Khaletskaya, A.B. Stroganov, N.I. Kubysheva, T.I. Eliseeva

**Affiliations:** Medical Resident, Department of Hospital Pediatrics; Privolzhsky Research Medical University, 10/1 Minin and Pozharsky Square, Nizhny Novgorod, 603005, Russia; Associate Professor, Department of Hospital Pediatrics; Privolzhsky Research Medical University, 10/1 Minin and Pozharsky Square, Nizhny Novgorod, 603005, Russia; Medical Resident, Department of Hospital Pediatrics; Privolzhsky Research Medical University, 10/1 Minin and Pozharsky Square, Nizhny Novgorod, 603005, Russia; Professor, Head of the Department of Children’s Diseases; Peoples’ Friendship University of Russia, 6 Miklukho-Maklaya St., Moscow, 117198, Russia; Medical Resident, Department of Hospital Pediatrics; Privolzhsky Research Medical University, 10/1 Minin and Pozharsky Square, Nizhny Novgorod, 603005, Russia; Consultant; City Clinical Hospital No.38, 22 Chernyshevskogo St., Nizhny Novgorod, 603000, Russia; Professor, Head of the Department of Hospital Pediatrics; Privolzhsky Research Medical University, 10/1 Minin and Pozharsky Square, Nizhny Novgorod, 603005, Russia; Associate Professor, Department of Faculty Surgery and Transplantology; Privolzhsky Research Medical University, 10/1 Minin and Pozharsky Square, Nizhny Novgorod, 603005, Russia; Senior Researcher, Research Laboratory “Clinical Linguistics”; Kazan Federal University, 18 Kremlyovskaya St., Kazan, Republic of Tatarstan, 420008, Russia; Professor, Department of Hospital Pediatrics; Privolzhsky Research Medical University, 10/1 Minin and Pozharsky Square, Nizhny Novgorod, 603005, Russia

**Keywords:** bronchial asthma, spirometry, obesity in children, nutritional status in children.

## Abstract

The potential mechanisms of bronchial asthma (BA) negative modification under the influence of obesity are currently being actively studied. However, at present, the effect of nutritional status on bronchial obstruction in children with BA cannot be considered established. In this regard, the **purpose of this work** was to study the relationship of spirometric parameters reflecting bronchial patency with nutritional status in children with asthma.

**Materials and Methods.:**

The study involved 54 patients with BA at the age of 8 to 17 years, 33 boys and 21 girls with different nutritional status. Assessment of nutritional status was carried out with the calculation of body mass index (BMI), relative body mass index (RBMI), and determination of body fat (% BF). Spirogram parameters were evaluated, including forced vital capacity (FVC), forced expiratory volume in 1 second (FEV1), FEV1/FVC ratio, maximum expiratory flow at the point of 25% loop flow-volume (MEF 25).

**Results.:**

Among the children examined, taking into account the BMI Z-score, 9.3% (5/54) had low body weight (group 1), 33% (18/54) had normal body weight (group 2), 31.5% (17/54) overweight (group 3), 25.9% (14/54) obesity (group 4). As the body weight increased, a statistically significant decrease in the FEV1/FVC ratio was observed, amounting to 84.6 [79.3; 90.0], 79.4 [76.6; 82.2], 74.6 [71.7; 77.5], 70.2 [67.0; 73.4]%, respectively, p=0.003; as well as a decrease in MEF 25 (% pred.), which amounted, respectively, to 95.6 [76.1; 115.2], 81.7 [71.4; 92.0], 56.3 [45.7; 66.9], and 48.4 [36.7; 60.1]%, p=0.003. A statistically significant negative relationship was found between indicators of nutritional status, including BMI, RBMI, % BF, and spirometry parameters reflecting bronchial patency, including FEV1/FVC ratio and MEF 25 (% pred.); all p<0.01.

**Conclusions.:**

Overweight and obesity in children with BA, estimated both by calculated methods with determination of BMI and RBMI and direct determination of body fat content, are accompanied by a statistically significant decrease in bronchial patency.

## Introduction

Bronchial asthma (BA) is a heterogeneous disease characterized by the presence of respiratory symptoms such as wheezing, shortness of breath, chest congestion, and cough that vary in time and intensity and are associated with reversible bronchial patency disorder [1]. The pathogenetic basis of BA is chronic allergic inflammation of the respiratory tract associated with bronchial hyperreactivity. The goal of BA therapy at the present stage is to achieve control over the symptoms, course, and risk factors of exacerbation of the disease, implemented in the course of basic anti-inflammatory therapy [1, 2]. It is believed that control of BA can be achieved in all patients, this is emphasized in the international conciliation documents, including GINA 2019 [1]. However, according to Braido et al. [3], up to 56% of patients do not have a proper level of disease control. One of the significant obstacles to achieving BA control is the presence of comorbid conditions [4–8]. In the list of common variants of comorbidity in recent years, close attention is focused on the combination of BA and obesity [9]. The prevalence of both diseases has increased significantly in recent years [10, 11]. It is believed that the combination of BA and obesity can contribute to the mutual aggravation of these diseases [12]. Currently, the potential mechanisms of negative modification of BA under the influence of obesity, including the adverse effects of overweight on the parameters of respiratory functions, are actively studied [13, 14]. The priority method of studying the functional features of the respiratory tract is the method of spirometry, which is the gold standard for assessing bronchial patency, included in the current recommendations for the management of patients with BA [1, 15—17].

However, there are currently conflicting data on the effect of overweight and obesity on spirometric parameters characterizing bronchial patency in patients with BA. So Forno and Celedón [18, 19] on the basis of the meta-analysis believe that the relationship between the most important spirometric index characterizing bronchial patency, namely, the forced expiratory volume in 1 second in percent predicted (FEV1 (% pred.)), and the body mass index (BMI), which is a screening method for assessing nutritional status, currently cannot be considered established. In a study by Kasteleyn et al. [20], obesity was shown to negatively affect lung function in adult patients with BA, manifested by a decrease in FEV1 and forced vital capacity (FVC) in obese patients compared to non-obese patients. Similar results were obtained by Somashekar et al. [21], who demonstrated a significant inverse correlation between the values of FEV1 (% pred.) and BMI in asthma patients aged 7–12 years — as BMI increased, there was a decrease in FEV1 (% pred.). At the same time in the work of Wang et al. [22] paradoxical results were obtained. The authors found that a higher BMI was statistically significantly associated with an increase in FEV1 and FVC, but only in girls. The study by Tantisira et al. [23] also found that in children with BA aged 5 to 12 years, the increase in BMI parameters is accompanied by an increase in FEV1 and FVC, but a decrease in the FEV1/FVC ratio. In work Yao et al. [13], the increase in BMI was associated by an increase in FEV1, FVC, but had a negative relationship with FEV1/ FVC. Thus, the data on the relationship between obesity and spirometric parameters characterizing bronchial patency are currently quite contradictory.

The existing contradictions may be due to the fact that the bulk of studies devoted to the study of the influence of obesity on spirometric indicators in children with BA, is used BMI as a criterion for assessing nutritional status according to WHO recommendations. But the same BMI values may correspond to different types of nutritional status in children of different ages and genders [11]. For example, a BMI of 17.0 in a boy of 11 years 1 month with average physical development (143 cm) will correspond to the median, a girl of 5 years 1 month (height 110 cm) — overweight (+1Z), and a girl of 17 years (height 163 cm) — protein-energy deficiency (–2Z). This complicates the formation of unified databases to study the relationship between nutritional status and respiratory parameters in patients with childhood BA and may be one of the reasons for obtaining conflicting results. To overcome these difficulties, we proposed a method for studying nutritional status in children with the introduction of the RBMI (relative body mass index) parameter, which is calculated as the ratio of patient BMI and median BMI values for a given age and sex, reflected in the WHO materials [11, 24].

In addition, it is obvious that BMI may not fully reflect the excess fat content in the body, because the increase in body weight may be due, for example, the increase in muscle mass in athletes, an increase in bone mineral density, other factors [25, 26]. In this regard, it seems appropriate to study the relationship of the parameters of external respiration not only with BMI, which is, in fact, a convenient screening method for assessing nutritional status, but also with direct indicators of body fat. Studies of this nature are isolated, and relate mainly to a cohort of adult patients. In particular, Sutherland et al. [27], Kamal et al. [28], Alaagib et al. [29], McLachlan et al. [30], Myung et al. [31].

In children, this issue is even less studied. We found three publications on the study of the potential impact of body fat on bronchial patency in children. A study by Kongkiattikul and co-authors [32] demonstrated the negative effect of excess fat on residual lung capacity in children. In the work of Mukherjee and Mukhopadhyay [33], a significant difference in the values of FVC, FEV1 was obtained between underweight, normal body weight subjects and overweight subjects. Body fat percentage (% BF) correlated with spirometric parameters more strongly than BMI.

Thus, the effect of nutritional status on bronchial patency in children with BA cannot be considered established. This makes it difficult to understand the mechanism of the negative impact of obesity and BA in children. In this regard, the present study aims to work the relationship of spirometric parameters reflecting bronchial patency with BMI, RBMI, and body fat in children with asthma.

## Materials and Methods

### Formation of the cohort of patients.

The study was conducted according to the Helsinki Declaration adopted in June 1964 (Helsinki, Finland) and revised in October 2000 (Edinburgh, Scotland). The study was approved by the Ethics Committee of Privolzhsky Research Medical University. Informed consent was obtained from the patients between 15 and 17 years old and from the parents of patients under the age of 15, according to the Federal Law “On the basis of health protection of citizens in the Russian Federation” of November 21, 2011, No.323.

A total of 54 children and adolescents aged from 8 to 17 years were examined, boys amounted for 61.1% (33/54), girls amounted for 38.9% (21/54), who were treated for atopic asthma in the Children’s City Clinical Hospital No.1 of Nizhny Novgorod, Russia in 2018–2019 were examined.

The inclusion criteria were a diagnosis of asthma in accordance with existing international and national conciliation instruments (GINA report, Global Strategy for Asthma Management and Prevention, 2016–2019) [1, 34]. The exclusion criteria were: the presence of acute infectious diseases and fever, diabetes, autoimmune disorders, primary immunodeficiency and cancer, oral glucocorticoids [35]. The diagnosis of BA and the disease severity were established by an attending doctor in accordance with recommendations valid at that period of time. Treatment of asthma was carried out in compliance with existing conciliation documents, taking into account modern therapeutic strategies [1, 2, 34].

### Objective measurement.

All children underwent general clinical examination, assessment of sensitization by skin tests to the main allergens, sensitization to which is typical for the Volga-Vyatka region of Russia [36]. Also, all patients were evaluated basic anthropometric indicators (height, body weight). Body weight was measured without shoes and without outwear, using scales with sensitivity up to 0.01 kg. Height measurements were performed using a height meter with a scale division up to 0.1 cm. Anthropometric parameters (height, body weight, and BMI) were evaluated according to WHO criteria [11].

The BMI (body weight/height^2^ (kg/m^2^)) and the RBMI we proposed earlier were also calculated [24, 36].

Children’s BMI was assessed based on their gender and age using Z-score criteria as recommended by WHO [37]. In accordance with these criteria for assessing BMI in this work, children were divided into 4 classes of nutritional status.

Group 1. Thinness. This group includes children with BMI values in the range of units from –1Z and less.

Group 2. Normal weight. This group includes children with median BMI values in the range of units from –1Z to +1Z on the Z-score scale.

Group 3. Overweight. This group includes children whose BMI exceeded the median BMI values by a range of units above +1Z but below +2Z.

Group 4. This group includes children whose BMI exceeded by +2Z and more.

Relative body mass index is the ratio of the individual BMI obtained to the median BMI for a given age and sex taken from the WHO materials. RBMI in contrast to BMI is an indicator that already contains an adjustment for the sex and age of the child [24, 36].

Body fat percentage was measured using a body composition monitor (Omron BF-214, Japan).

The quantitative assessment of BA status was carried out using the Asthma Control Questionnaire-5 (ACQ-5). With the ACQ-5 score below 0.75, BA was considered fully controlled, with the ACQ-5 scores from 0.75 to 1.5 — partially controlled, and the score above 1.5 indicated uncontrolled BA [38].

### Assessment of respiratory function.

Spirometric studies were conducted using the MasterScreen Pneumo spirometer (Jaeger, Germany) following the existing international guidelines. Evaluated FVC, FEV1, maximum expiratory flow at the point of 25% (MEF 25) loop flow-volume; the data were recorded both in absolute values (L/s) and in relative units — percent of the designed values (% pred.) taking into account gender, age, and anthropometric indices of the child. The FEV1/FVC index was also evaluated [39].

### Statistical analysis.

The study was pilot, and therefore the calculation of the required sample size was not carried out. Statistical analysis was performed using the Statgraphics Centurion software package v.16.1.17. Data are presented as Me [Q1; Q3], where Me stands for the median, [Q1; Q3] is the interquartile range.

When checking the sample for normality, the standardized skewness and standardized kurtosis were calculated for quantitative characteristics of the sample. If these calculated values of standardized asymmetry and standardized excess are outside the range from –2 to +2, then the considered quantitative samples are considered different from normal. Differences between the two groups were determined using Student’s t-test to compare the average values of two samples (for samples that had a normal distribution) and using the Wilcoxon (Mann–Whitney) W-test to compare the medians of two samples (for samples that had an excellent distribution from normal). ANOVA analysis of variance (criterion F) was used to compare the average values of several groups (samples with normal distribution), and the Kruskal–Wallis test (KWT criterion) was used to compare medians of several groups (samples with a distribution other than normal). The relationship between spirometric parameters and indicators reflecting the nutritional status of patients (BMI, RBMI, % BF) was evaluated using Spearman’s rank correlation. Differences were considered statistically significant at p<0.05.

## Results

### Clinical characteristics of patients.

The analysis was carried out both in the General cohort and separately in boys and girls. The median age of children was 13.41 [12.67; 14.14] years, boys and girls were comparable in age and ACQ-5 scores (Table 1). Anthropometric parameters of boys, including height and body weight, were statistically significantly higher than those of girls. Indirect indices characterizing nutritional status, including BMI and RBMI, in girls and boys in the study sample were also comparable. It is worth noting that girls had higher body fat percentages than boys, apparently due to sexual dimorphism. Muscular tissue mass is known to be of greater importance for the body mass increase in boys whereas in girls it depends on the fraction of the adipose tissue [40].

**Table 1:** Clinical and functional characteristics of examined children with bronchial asthma, Me [Q1; Q3]

Parameters	The total cohort (n=54)	Boys (n=33)	Girls (n=21)	Statistics between of difference groups
Age (years)	13.41 [12.67; 14.14]	13.58 [12.71; 14.44]	13.14 [11.73; 14.56]	W=334.0; p=0.83
Height (cm)	163.4 [159.8; 167.1]	167.2 [162.5; 171.9]	157.5 [152.3; 162.7]	W=199.0; p=0.009
Weight (kg)	60.42 [56.42; 64.41]	64.53 [59.98; 69.07]	53.96 [47.0; 60.93]	W=186.5; p=0.005
BMI	22.44 [21.40; 23.48]	23.06 [21.87; 24.25]	21.47 [19.50; 23.44]	t=1.51; p=0.14
RBMI	1.20 [1.14; 1.27]	1.23 [1.14; 1.31]	1.17 [1.06; 1.28]	W=293.0; p=0.34
Body fat percentage	20.42 [17.76; 23.09]	16.48 [13.94; 19.02]	26.61 [21.96; 31.27]	W=545.0; p=0.0004
ACQ-5 (points)	0.88 [0.63; 1.13]	0.83 [0.54; 1.13]	0.96 [0.47; 1.45]	W=356.5; p=0.86
FVC (L)	4.04 [3.73; 4.34]	4.42 [4.03; 4.81]	3.43 [3.06; 3.80]	t=3.52; p=0.0009
FVC (% pred.)	111.0 [107.2; 114.1]	110.2 [106.0; 114.7]	111.0 [104.5; 117.6]	t=–0.23; p=0.82
FEV1 (L)	3.07 [2.81; 3.33]	3.39 [3.04; 3.75]	2.56 [2.29; 2.83]	W=166.5; p=0.001
FEV1 (% pred.)	100.0 [95.1; 105.9]	101.1 [95.3; 107.0]	98.2 [89.9; 106.5]	t=0.61; p=0.54
FEV1/FVC (%)	76.3 [73.2; 79.3]	76.4 [73.4; 79.4]	75.4 [70.6; 80.2]	t=0.39; p=0.70
MEF 25 (L/s)	1.40 [1.17; 1.63]	1.60 [1.28; 1.93]	1.08 [0.80; 1.35]	W=205.0; p=0.01
MEF 25 (% pred.)	66.2 [57.1; 76.3]	72.1 [59.6; 84.5]	57.4 [43.1; 71.6]	W=258.5; p=0.12

Spirometry parameters reflecting absolute values of external respiration, including FVC (L), FEV1 (L), MEF 25 (L/s) in boys were higher than in girls. At the same time, these indicators of spirometry in relative terms (% pred.) did not have significant gender differences, which may indicate the comparability of bronchial patency in comparison with the proper indicators in girls and boys in this sample.

The body fat and RBMI content of children grouped according to the Z-score BMI system (WHO) was expected to increase statistically significantly as the BMI class increased, both in the sample as a whole and in the boys and girls group (Table 2).

**Table 2:** Body fat content and RBMI in children with bronchial asthma taking into account nutritional status (BMI according to the Z-score system, WHO), Me [Q1; Q3]

Parameters	Thinness	Normal weight	Overweigh	Obesity	Statistics between of difference groups
***The total cohort (n=54)***					
Number of patients	5	18	17	14	—
RBMI	0.86 [0.80; 0.90]	1.01 [0.90; 1.20]	1.25 [1.20; 1.30]	1.52 [1.40; 1.70]	KWT=47.19; p<0.0001
Body fat percentage	9.06 [6.00; 11.90]	18.64 [6.40; 36.60]	21.58 [6.90; 39.70]	25.36 [11.30; 45.10]	KWT=13.26; p=0.004
***Boys (n=33)***					
Number of patients	3	8	12	10	—
RBMI	0.87 [0.80; 0.90]	0.98 [0.90; 1.10]	1.25 [1.20; 1.30]	1.51 [1.40; 1.70]	F=82.12; p<0.0001
Body fat percentage	7.50 [6.0; 8.90]	12.90 [6.40; 19.9]	18.32 [6.90; 27.30]	19.83 [11.30; 34.0]	F=4.19; p=0.01
***Girls (n=21)***					
Number of patients	2	10	5	4	—
RBMI	0.85 [0.80; 0.90]	1.03 [0.90; 1.20]	1.26 [1.20; 1.30]	1.55 [1.40; 1.60]	F=45.10; p<0.0001
Body fat percentage	11.40 [10.90; 11.90]	23.23 [12.20; 36.60]	29.42 [18.50; 39.70]	39.18 [33.40; 45.10]	F=8.41; p=0.001

### Spirometric indicators characterizing bronchial patency in children with BA taking into account the nutritional status of patients.

**Figure 1:**
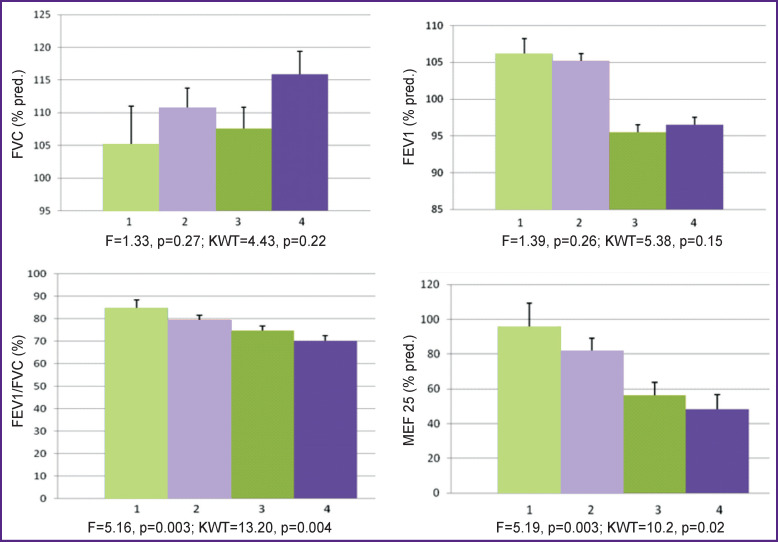
Spirometric parameters in children with bronchial asthma taking into account their nutritional status: *1* — thinness; *2* — normal weight; *3* — overweight; *4* — obesity

We have found that in the total cohort of patients with asthma have a progressive statistically significant decrease FEV1/FVC index and MEF 25 (% pred.) with increasing BMI class (Table 3, Figure 1). In addition, there is a trend to an increase in FVC (% pred.) and lower FEV1 (% pred.) with increasing BMI class, however, these differences are not statistically significant.

**Table 3:** Spirometric parameters in children with different classes of nutritional status (BMI according to the Z-score system, WHO), Me [Q1; Q3]

Parameters	Thinness	Normal weigh	Overweigh	Obesity	Statistics between of difference groups
***The total cohort (n=54)***					
Number of patients	5	18	17	14	—
FVC (% pred.)	105.1 [96.7; 113.5]	110.7 [106.2; 115.1]	107.6 [106.2; 115.1]	115.9 [110.9; 120.9]	F=1.33; p=0.27
FEV1 (% pred.)	106.1 [95.4; 116.9]	105.2 [99.6; 110.9]	95.5 [89.7; 101.4]	96.5 [90.1; 103.0]	F=1.39; p=0.26
FEV1/FVC (%)	84.6 [79.3; 90.0]	79.4 [76.6; 82.2]	74.6 [71.7; 77.5]	70.2 [67.0; 73.4]	F=5.16; p=0.003
MEF 25 (% pred.)	95.6 [76.1; 115.2]	81.7 [71.4; 92.0]	56.3 [45.7; 66.9]	48.4 [36.7; 60.1]	KWT=10.2; p=0.02
***Boys (n=33)***					
Number of patients	3	8	12	10	—
FVC (% pred.)	112.0 [101.4; 122.5]	112.8 [106.3; 119.2]	104.2 [98.9; 109.4]	114.9 [109.0; 120.7]	F=1.50; p=0.23
FEV1 (% pred.)	117 [105.6; 128.9]	114 [106.5; 120.7]	92.7 [86.9; 98.5]	96.5 [90.1; 102.9]	F=5.30; p=0.005
FEV1/FVC (%)	86.3 [80.9; 91.7]	83.3 [80.0; 86.7]	74.3 [71.6; 77.0]	70.3 [67.4; 73.3]	F=8.67; p=0.0003
MEF 25 (% pred.)	115.3 [92.8; 137.9]	101.2 [87.4; 15.0]	58.4 [47.1; 69.7]	52.1 [39.8; 64.5]	KWT=14.02; p=0.003
***Girls (n=21)***					
Number of patients	2	10	5	4	—
FVC (% pred.)	94.8 [80.4; 109.4]	108.9 [102.5; 115.4]	115.8 [102.5; 115.4]	118.5 [108.3; 128.8]	F=1.59; p=0.23
FEV1 (% pred.)	89.5 [68.9; 110.0]	98.6 [61.6; 89.4]	102.3 [89.3; 107.8]	96.6 [82.0; 111.1]	F=0.22; p=0.88
FEV1/FVC (%)	82.2 [70.1; 93.7]	76.2 [71.1; 81.4]	75.3 [68.1; 82.6]	69.9 [61.8; 78.0]	F=0.62; p=0.61
MEF 25 (% pred.)	66.0 [32.5; 99.5]	66.1 [51.1; 81.1]	51.2 [30.0; 72.4]	39.0 [15.3; 62.7]	F=0.81; p=0.51

In boys with increasing BMI class, a statistically significant decrease of the FEV1/FVC index, MEF  25 (% pred.), and FEV1 (% pred.) was revealed. In girls, statistically significant changes in FVC (% pred.), FEV1 (% pred.), MEF 25 (% pred.), and the FEV1/FVC index in the groups distinguished with consideration of BMI value has not been established, although their trends generally correspond to those in the total cohort, and among boys (see Table 3).

### Relationship of nutritional status and bronchial patency in patients with BA.

We evaluated the relationship between spirometry parameters reflecting bronchial patency and indicators characterizing the nutritional status of patients (Table 4). The relationship of the relative values of FVC (% pred.), FEV1 (% pred.) with the parameters characterizing the nutritional status of patients, including BMI, RBMI, and % BF content in this sample of patients with BA has not been established. However, there was noted a tendency to the increase of the relative parameters of FVC (% pred.), p=0.07, with the increase of BMI.

**Figure 2:**
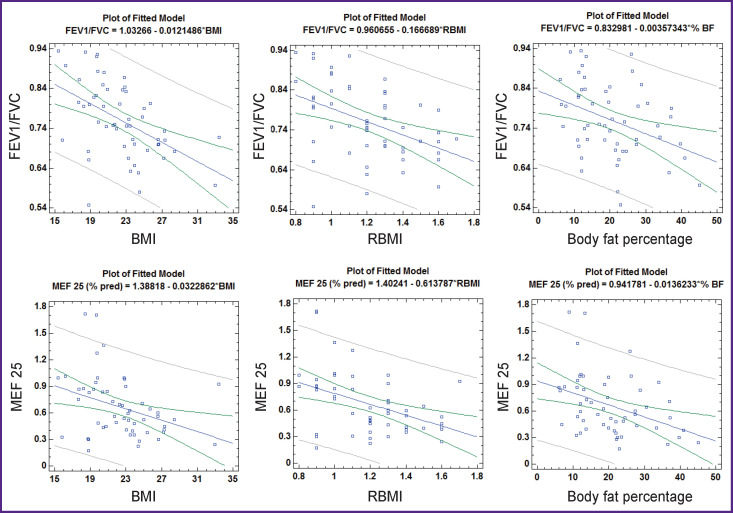
Correlation of indicators reflecting nutritional status with the FEV1/FVC index and MEF 25, whole population (n=54)

The most important from the clinical point of view is the identification of a statistically significant negative relationship of all considered parameters characterizing nutritional status with relative values of MEF 25 (% pred.) and with the FEV1/FVC index (see Table 4, Figure 2). Given that the FEV1/FVC index is currently regarded as an important spirometric marker characterizing bronchial patency, and MEF 25 (% pred.) as a spirometric marker of small bronchial patency, it should be noted that as BMI, RBMI and body fat increase, there is a decrease in bronchial patency, including patency in the small airway.

**Table 4:** Correlation of relative spirometry parameters and anthropometric data characterizing the nutritional status in children with bronchial asthma

Parameters	FVC	FEV1	FEV1/FVC	MEF 25
BMI	0.25 (0.07)	–0.18 (0.21)	–0.50 (0.0001)	–0.36 (0.008)
RBMI	0.22 (0.11)	–0.18 (0.18)	–0.43 (0.001)	–0.43 (0.001)
Body fat percentage	0.09 (0.52)	–0.22 (0.11)	–0.38 (0.005)	–0.39 (0.004)

The data are presented as r (p), where r is the correlation coefficient, p is the level of statistical significance.

**Figure 3:**
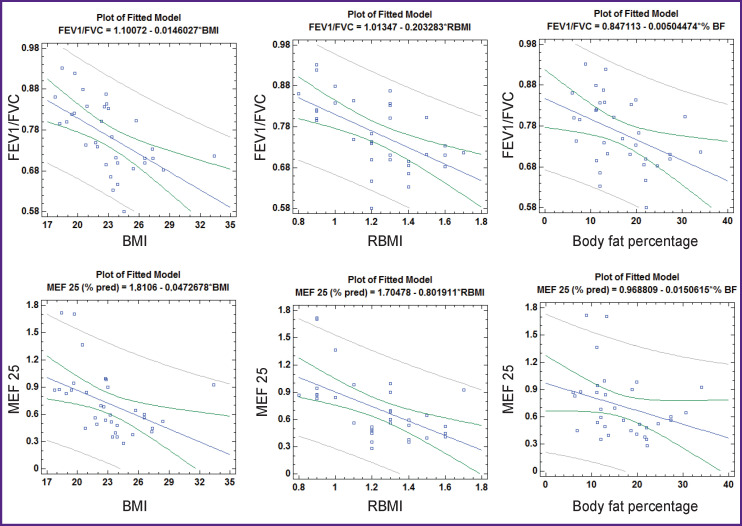
Correlation of indicators reflecting nutritional status with the FEV1/FVC index and MEF 25 in boys with bronchial asthma

**Figure 4:**
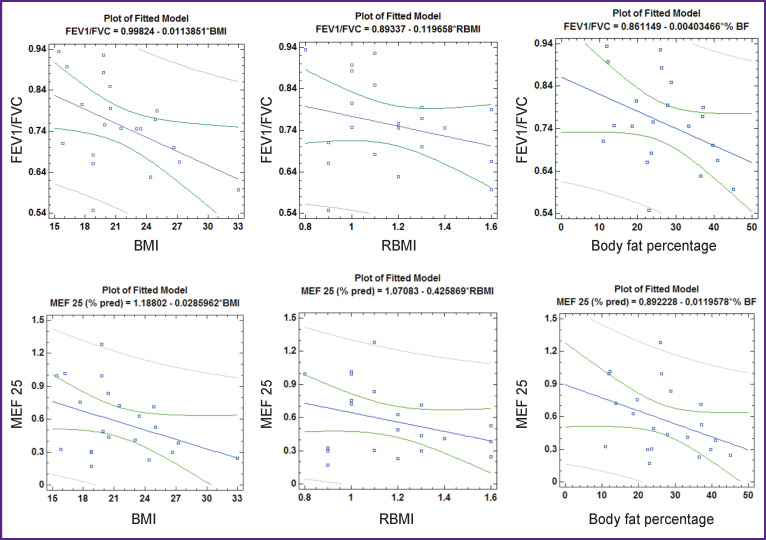
Correlation of indicators reflecting nutritional status with the FEV1/FVC index and MEF 25 in girls with bronchial asthma

When considering the relationship of spirometric parameters and nutritional status of children with BA, taking into account gender differences, the following is established (Table 5, Figures 3 and 4). In boys, all indicators characterizing nutritional status show a statistically significant inverse correlation with the FEV1/ FVC index. BMI and RBMI in boys are also negatively correlated with MEF 25, (% pred.), and RBMI with FEV1 (% pred.).

**Table 5:** Correlation of spirometry parameters and indicators reflecting nutritional status

Parameters	FVC	FEV1	FEV1/FVC	MEF 25
***Boys (n=33)***				
BMI	0.07 (0.72)	–0.36 (0.04)	–0.58 (0.0005)	–0.45 (0.008)
RBMI	0.09 (0.60)	–0.38 (0.03)	–0.58 (0.0004)	–0.55 (0.0008)
Body fat percentage	–0.07 (0.70)	–0.31 (0.08)	–0.42 (0.01)	–0.31 (0.08)
***Girls (n=21)***				
BMI	0.48 (0.03)	–0.02 (0.94)	–0.47 (0.03)	–0.40 (0.08)
RBMI	0.42 (0.06)	0.07 (0.77)	–0.27 (0.24)	–0.33 (0.15)
Body fat percentage	0.25 (0.28)	–0.11 (0.63)	–0.39 (0.08)	–0.39 (0.08)

The data are presented as r (p), where r is the correlation coefficient, p is the level of statistical significance.

In girls, in contrast to boys, there was also a direct relationship between BMI and RBMI with FVC (% pred.). In addition, a clear negative correlation has been established between BMI and the FEV1/FVC index (r=–0.47; p=0.03) which was typical for boys as well. There were no statistically significant correlations of body fat content with spirometric parameters in girls, but there is a clear tendency to the inverse correlation between the content of % BF with the FEV1/FVC index (r=–0.39; p=0.08) and with MEF 25 (% pred.) (r=–0.39; p=0.08) (see Figure 4). This is in line with the trends typical for boys and girls and the sample as a whole.

## Discussion

The study was the first to compare several characteristics of nutritional status, including assessment of BMI, RBMI, and body fat, with spirometric parameters characterizing bronchial patency in children with BA.

Our study established clear negative relationships between BMI and FEV1/FVC, the most important spirometric index characterizing bronchial patency. These patterns were typical for both the total cohort of patients (r=–0.50; p=0.0001), which is consistent with the data of Yao et al. [13], Duncan et al. [41], and separately for boys (r=–0.58; p=0.0005) and girls (r=–0.47; p=0.03). Thus, our data indicate that an increase in BMI, which is an indirect sign of overweight and obesity, can be considered as a predictor of worsening bronchial patency in patients with BA regardless of the sex of the child.

It is important to note that the negative impact of overweight and obesity on bronchial patency in the total cohort of patients in our study was also demonstrated by the use of such tools for assessing nutritional status, as RBMI and determination of body fat.

Our findings on the presence of a statistically significant inverse relationship between % BF and FEV1/ FVC ratio in the overall cohort of children with BA are not entirely consistent with some available literature data. For example, Wang and co-authors [22] found no statistically significant relationship between body fat and lung function. However, it should be noted that in the study of Wang et al., the analysis of the relationship of spirometric parameters with BMI and % BF was carried out in the general population, and no separate analysis was performed for patients with BA. Perhaps, in order to study in depth the effect of obesity, in particular body fat, on lung function in asthma patients, it is not the general population that should be considered, but rather patients with BA. It is possible that in conditions of persistent allergic inflammation in the respiratory tract in patients with asthma, modulation of the systemic inflammatory response by adipose tissue may selectively lead to negative modification of bronchial patency mainly in asthmatic airway, which requires further detailed research [42].

It is obvious that there may be various pathogenetic mechanisms of the influence of obesity on the physiology of the lungs, including, inter alia, an imbalance of pro- and anti-inflammatory cytokines. Adipokines are known to be involved in bronchial inflammation and hyperreactivity, which can also worsen the course of BA [43]. On the other hand, there is circumstantial evidence suggesting that BA itself may have influenced changes in nutritional status. And this is due not only to changes in the level of physical activity in connection with the disease and the reception of exogenous glucocorticoids, but also with the possibility of local synthesis of alphamelanocyte stimulating hormone, respectively, and its predecessor-adrenocorticotropic hormone (ACTH) [44]. The role of local synthesis of ACTH and the possibility of hypercortisolemia against this background have yet to be evaluated, especially given the developing resistance to glucocorticoids against obesity [45]. It should be noted that the results of our previous study showed a tendency to increase the proportion of overweight children as the course of asthma becomes heavier [24].

It is also important to note that in our study in the total cohort of patients revealed a negative relationship between the % BF and MEF 25, which is considered as an important characteristic of patency of small airway [46]. The increase in the likelihood of obstruction of the small airway as the percentage of body fat increases may be considered as one of the potential reasons preventing the achievement of control in patients with BA associated with overweight and obesity.

In our work, no statistically significant relationship between the nutritional status of patients and FVC was revealed. However, it is noteworthy that in general, in the study sample there is a trend to increase FVC as BMI increases. This somewhat contradicts the available data that for patients with obesity is characterized by a decrease in FVC, due to mechanical reasons due to the limiting effect of the fat layer on the pulmonary volumes [47, 48], but is consistent with data from other authors, e.g. Wang et al. [22], which found that higher BMI was statistically significantly associated with increased FVC in girls. Further studies may be able to clarify this contradiction, including the analysis of spirometric indicators in children with BA, taking into account the dynamics of body weight and obesity. For example, in Real et al. [49] it has been demonstrated that FVC can increase in women synchronously with BMI to BMI=25 kg/m^2^ (the boundary of normal and overweight in adult women), but with further increase in BMI, the authors recorded a decrease in FVC. The authors even conclude that a BMI of 24–25 is optimal for pulmonary function [50]. A possible explanation for the seemingly paradoxical increase in lung capacity as BMI increases is the potential increase in musculoskeletal mass, also contributing to the increase in BMI in some patients, especially in child athletes [51].

It should be noted that a direct relationship between increased BMI and increased FVC has been noted in several studies. So in the work of Yao and co-authors [13], it was found that the increase in BMI in children was statistically significantly associated with an increase in FVC, FEV1, but with a decrease in the FEV1/FVC ratio. The authors explain the findings as a consequence of dysanapsis, a disproportionate growth of the respiratory tract relative to the lung parenchyma. At the same time, the size of the lungs in obese children is larger than in children with normal weight, but the size of the airways has not yet grown in proportion to the size of the lungs [52].

Several other studies have been conducted evaluating the association of obesity with lung function in different populations of children [53–55]. Han et al. [53] demonstrated that increased BMI is associated with higher FVC and FEV1 and lower FEV1/FVC ratio among a sample population of children without asthma. Similarly, Cibella et al. [54] the study showed that weight positively correlated with FVC and FEV1, but negatively correlated with the FEV1/FVC ratio in adolescents. Similar results have been reported in children with asthma [23].

Other studies either failed to find a link between obesity rates and lung function in children or reported conflicting results [56, 57].

Thus, it seems that overweight and obesity can form a special phenotype of external respiration in both patients with BA and healthy ones, consisting in a disproportionate increase in FVC in combination with a decrease in bronchial patency, recorded by a decrease in FEV1/FVC ratio. However, in patients with asthma, perhaps it may contribute to violations of bronchial obstruction, characteristic of this disease.

Minor gender differences in the relationship between bronchial patency and nutritional status, obtained in our work, can be explained both by the number of boys and girls studied, and by different laws of distribution of adipose tissue in them. With an equivalent pre-pubertal BMI, girls have a higher body fat content than boys. It is also worth noting that the results may be affected by fluctuations in progesterone and estrogen levels during menarche [58].

The limitation of our study is the lack of analysis of sexual development of adolescents and menstrual cycle in girls. In the literature there is evidence of the association of more severe asthma with BMI in girls with early menarche [59]. Real et al. showed synergistic effect on FVC and FEV1 oligomenorrhea and BMI [49, 50].

Thus, in children with BA, there is a statistically significant negative relationship of spirometric parameters characterizing bronchial patency, especially FEV1/FVC ratio, both with the calculated parameters characterizing the nutritional status of patients, including BMI and RBMI, and with the % BF. Therefore, we believe that in the management of patients with childhood BA, both the calculation of BMI and the determination of body fat should be considered as a necessary component of an objective examination. However, it is worth noting that the WHO criteria for determining obesity and overweight are based only on BMI, and are not designed to determine the % BF in children.

## Conclusion

Overweight and obesity in children with BA, estimated both by calculated methods with determination of BMI and RBMI and direct determination of body fat content, are accompanied by a statistically significant decrease in bronchial patency, estimated by the FEV1/FVC index, as well as a decrease in bronchial patency at the level of small bronchi, estimated by MEF 25 (L/s) and MEF  25 (% pred.). The phenotype of children suffering from BA and obesity requires deeper study to address practical issues related to the diagnosis, treatment, and effective control of BA. An increase in BMI, an indirect indicator of obesity, is a predictor of worsening bronchial patency in patients with BA regardless of the sex of the child.
